# Real-world treatment patterns, survival outcomes, and health care resource utilization for locally advanced or metastatic urothelial carcinoma in Spain

**DOI:** 10.1007/s12094-024-03734-8

**Published:** 2024-10-04

**Authors:** Javier Puente, Alvaro Pinto, Maria José Mendez-Vidal, Xavier García del Muro, Pablo Maroto, Sergio Vazquez, Raquel Luque-Caro, Urbano Anido, Torsten Strunz-McKendry, Anil Upadhyay, Jose Montes, Aurora Ortiz Nuñez, Judit González Portela, Daniel Castellano

**Affiliations:** 1 Medical Oncology Department, Hospital Clínico San Carlos, Instituto de Investigación Sanitaria del Hospital Clínico San Carlos (IdISSC), CIBERONC, Calle del Prof Martín Lagos, S/N, 28040 Madrid, Spain; 2 Servicio de Oncología Médica, Hospital Universitario La Paz, Madrid, Spain; 3 Medical Oncology Department, Maimonides Institute for Biomedical Research of Córdoba (IMIBIC) Hospital Universitario Reina Sofía, Cordoba, Spain; 4 Institut Català d’Oncologia, IDIBELL. University of Barcelona, Barcelona, Spain; 5 Servicio de Oncología Médica, Hospital de La Santa Creu i Sant Pau, Barcelona, Spain; 6 Servicio de Oncología Médica, Hospital Universitario Lucus Augusti, Lugo, Spain; 7 Servicio de Oncología Médica, Hospital Universitario Virgen de Las Nieves, Instituto de Investigación Biosanitaria Ibs. Granada, Granada, Spain; 8 Servicio de Oncología Médica, Hospital Universitario de Santiago, Santiago, Spain; 9 Astellas Pharma Europe Ltd, Addlestone, Surrey UK; 10 Effice Research, Madrid, Spain; 11 Medical Oncology Department, Hospital Universitario, 12 de Octubre, Madrid, Spain

**Keywords:** Chemotherapy, Medical records, Metastatic neoplasm, Observational study, Urinary bladder neoplasms

## Abstract

**Purpose:**

Real-world evidence on locally advanced or metastatic urothelial carcinoma (la/mUC) management in Spain is limited. This study describes patient characteristics, treatment patterns, survival, and health care resource utilization (HCRU) in this population.

**Methods/patients:**

This retrospective observational study included all adults with a first diagnosis/record of la/mUC (index date) from January 2015 to June 2020 at nine university hospitals in Spain. Data were collected up to December 31, 2020 (end of study), death, or loss to follow-up. Patient characteristics, treatment patterns, median overall survival (OS) and progression-free survival (PFS) from index date (Kaplan–Meier estimates), and disease-specific HCRU were described.

**Results:**

Among 829 patients, median age at diagnosis was 71 years; 70.2% had ≥ 1 comorbidity, and 52.5% were eligible for cisplatin. Median follow-up was 12.7 months. Most (84.7%) patients received first-line systemic treatment; of these, 46.9% (*n* = 329) received second-line and 16.6% (*n* = 116) received third-line therapy. Chemotherapy was the most common treatment in all lines of therapy, followed by programmed cell death protein 1/ligand 1 inhibitors. Median (95% confidence interval) OS and PFS were 18.8 (17.5–21.5) and 9.9 (8.9–10.5) months, respectively. Most patients required ≥ 1 outpatient visit (71.8%), inpatient admission (56.6%), or emergency department visit (56.5%).

**Conclusions:**

Therapeutic patterns were consistent with Spanish guideline recommendations. Chemotherapy had a role in first-line treatment of la/mUC in Spain during the study period. However, the disease burden remains high, and new first-line treatments recommended in the latest European guidelines should be made available to patients in Spain.

**Supplementary Information:**

The online version contains supplementary material available at 10.1007/s12094-024-03734-8.

## Introduction

Bladder cancer is the 10th most frequently diagnosed cancer worldwide and the fifth most commonly diagnosed in Spain, with an estimated incidence of 21,694 cases by 2023 [1, 2]. At initial presentation, bladder cancers can be classified as non–muscle-invasive, muscle-invasive, or metastatic cancer [3]. Urothelial carcinoma (UC) is the predominant type of bladder cancer [4]. The Spanish Society of Medical Oncology estimates approximately 30% of new UC cases are diagnosed in the late stage every year [5].

Historically, first-line standard of care for patients with locally advanced or metastatic UC (la/mUC) is cisplatin-based combination chemotherapy [6]. For patients with la/mUC considered ineligible for cisplatin-based chemotherapy (≈50%), first-line carboplatin-based regimens are preferred [6, 7]. Pembrolizumab, a programmed cell death protein 1 (PD-1) inhibitor, and atezolizumab, a programmed death ligand 1 (PD-L1) inhibitor, are also first-line monotherapy options for patients ineligible for cisplatin whose tumors express PD-L1 [6]. Avelumab (approved in 2021 by the European Medicines Agency [8]) is recommended for maintenance therapy in a subset of patients without disease progression after first-line platinum-based chemotherapy [6]. For patients with disease progression after platinum-based chemotherapy, PD-1/L1 inhibitors are the standard option; erdafitinib is an alternative for patients with alterations in *FGFR* [6], and patients may receive vinflunine or taxane-based chemotherapy when other options are unavailable [6]. Enfortumab vedotin (EV) is recommended as an alternative to chemotherapy for tumors that have relapsed after first-line immunotherapy and is the standard of care for patients with disease progression after first-line chemotherapy and maintenance avelumab [6]. The 2024 European Society for Medical Oncology guidelines now recommend EV plus pembrolizumab as the new standard of care in first-line advanced or mUC, regardless of cisplatin eligibility [9]. Patients who are not able to receive EV plus pembrolizumab should be treated with (1) if cisplatin-eligible, nivolumab plus up to 6 cycles of gemcitabine–cisplatin or (2) up to 6 cycles of platinum-based chemotherapy (gemcitabine + cisplatin or carboplatin) followed by maintenance avelumab (for non-progressing tumors) [9].

Real-world evidence about epidemiology, treatment patterns, and health care resource utilization (HCRU) is lacking among patients with la/mUC in Spain. The study presented here used real-world data to provide information about patient characteristics, treatment patterns, survival, and HCRU in patients with la/mUC in Spain from 2015 to 2020.

## Patients and methods

### Study design and patients

This retrospective observational study was conducted using electronic medical records from nine geographically representative university hospitals in Spain. The study period was from January 1, 2015, to December 31, 2020. All adult (age ≥ 18 years) patients with a first diagnosis/record of la/mUC between January 1, 2015, and June 30, 2020 (inclusion period), were included in the study cohort; this allowed for a 6-month minimum follow-up period (Supplemental Fig. 1). Patients were required to have received a histologically or cytologically confirmed diagnosis of la/mUC; those with urachus carcinoma or other nonurothelial cancers were excluded. The index date was the date of first la/mUC record/diagnosis during the inclusion period. Data were collected from index date up to December 31, 2020 (end of study period), death, or loss to follow-up, whichever occurred first. An independent ethics committee reviewed and approved the study. Given the retrospective nature of the study, informed consent was not required.

### Data sources and measurement

Data were collected in a web-based electronic data capture system (ReseaArch, Madrid, Spain). Each investigator was trained to ensure accuracy and consistency across sites and to reduce the risk of information bias. Study data were accessible to authorized personnel alone and patient anonymity was maintained. Use of the electronic case report form included automatic data checks at the time of data entry to ensure data were consistent, complete, and coherent.

### Statistical methods

Patient demographics and clinical characteristics, including eligibility for cisplatin-based chemotherapy, were described for the study population. Ineligibility for cisplatin-based chemotherapy was aligned with Spanish guidelines and determined based on the presence of at least one of following criteria: performance status (PS) of two or higher (Eastern Cooperative Oncology Group [ECOG]) or 60–70% (Karnofsky), creatinine clearance less than 60 ml/minute, grade two or higher audiometric hearing loss, grade two or higher peripheral neuropathy, or New York Health Association class III heart failure [6, 7].

Treatment patterns, survival, and HCRU associated with la/mUC were also described. Treatment patterns included treatment type(s) received during the follow-up period (eg, systemic therapy, radiotherapy, surgery); lines of systemic therapy received; treatment regimen received each line; and treatment sequencing of systemic therapy. The number and percentage of patients and line setting for each regimen were reported. Median overall survival (OS) and real-world progression-free survival (PFS) from index date were determined using Kaplan–Meier methods. Median OS was estimated overall and also stratified by first-line therapy received and eligibility for cisplatin-/platinum-based chemotherapy. Platinum ineligibility was determined based on the presence of at least one of the following criteria: ECOG PS of three or higher, creatinine clearance less than 30 ml/minute, grade two or higher peripheral neuropathy, New York Health Association class III heart failure, or a combination of ECOG PS of two and creatinine clearance less than 30 ml/minute [10]. Progression was defined as an increase in number or type of tumor, start of new treatment, or other diagnosis signifying progression, accounting for censoring. Patients who did not show progression were censored on the date of the last assessment of disease progression. Time to progression was defined as the time from index date to progression; patients who died prior to progression were censored on the date of death. Disease-specific HCRU was summarized from index date to end of study period, death, or loss to follow-up. It included the number of outpatient appointments, inpatient admissions, and emergency department (ED) visits per patient and the numbers per patient per month.

Descriptive statistics were used for continuous and categorical variables. All statistical analyses were performed with Microsoft Excel (Microsoft, Redmond, WA) and SAS version 9.4 (SAS Institute, Cary, NC).

## Results

### Study population and patient characteristics

A total of 829 patients were included in the study population. The median follow-up time was 12.7 months. Median patient age at la/mUC diagnosis was 71 years, and 80.1% of patients were men (Table 1). Among patients for whom smoking history was available (*n* = 696), 75.0% were current or former smokers. Most (70.2%) patients had at least one comorbidity; the most frequently reported comorbidities were cardiovascular disease (53.6%), respiratory disorders (27.5%), and diabetes (15.7%). The primary tumor site was the bladder (84.0%), and the most common initial UC diagnosis was la/mUC (42.1%). Most (80.2%) patients had metastases; of these 665 patients, 55.8% (*n* = 371) had metastases at visceral sites. Among patients with ECOG PS values reported (*n* = 625), most (78.7%) had an ECOG PS of zero or one; 52.5% of patients were eligible for cisplatin-based chemotherapy.

**Table 1:** Patient characteristics and clinical history at date of diagnosis of locally advanced or metastatic urothelial carcinoma

Variable	Follow-up cohort *N* = 829	1L treated *N* = 702	1L not treated *N* = 127
Age at diagnosis, years			
Median (range)	71 (63–77)	70 (62–76)	75 (68–82)
Mean (SD)	69.9 (10.0)	69.1 (9.8)	74.1 (10.4)
Male sex	664 (80.1)	558 (79.5)	106 (83.5)
Smoking	*n* = 696	*n* = 591	*n* = 105
Yes	172 (24.7)	146 (24.7)	26 (24.8)
Prior history	350 (50.3)	308 (52.1)	42 (40.0)
Never	174 (25.0)	137 (23.2)	37 (35.2)
Comorbidity	582 (70.2)	479 (68.2)	103 (81.1)
Cardiovascular disease	444 (53.6)	367 (52.3)	77 (60.6)
Respiratory disorder	228 (27.5)	171 (24.4)	57 (44.9)
Diabetes	130 (15.7)	111 (15.8)	19 (15.0)
Renal disease	63 (7.6)	54 (7.7)	9 (7.1)
Hepatic disease	44 (5.3)	38 (5.4)	6 (4.7)
Charlson Comorbidity Index	*n* = 119	*n* = 99	*n* = 20
Mean (SD)	7.6 (3.0)	7.7 (3.0)	6.8 (2.9)
Primary tumor site			
Bladder	696 (84.0)	583 (83.0)	113 (89.0)
Urethra	56 (6.8)	53 (7.5)	3 (2.4)
Upper urinary tract	52 (6.3)	45 (6.4)	7 (5.5)
Kidney	7 (0.8)	6 (0.9)	1 (0.8)
Renal pelvis	3 (0.4)	3 (0.4)	0 (0.0)
Other/unknown	15 (1.8)	12 (1.7)	3 (2.4)
Type of first diagnosis			
Non–muscle-invasive UC/BC	169 (20.4)	149 (21.2)	20 (15.7)
Muscle-invasive UC/BC	311 (37.5)	259 (36.9)	52 (40.9)
Locally advanced or metastatic UC	349 (42.1)	294 (41.9)	55 (43.3)
Diagnosis of locally advanced or metastatic UC			
Locally advanced	164 (19.8)	118 (16.8)	46 (36.2)
Metastases	665 (80.2)	584 (83.2)	81 (63.8)
Lymph node	375 (45.2)	335 (47.7)	40 (31.5)
Bone	151 (18.2)	133 (18.9)	18 (14.2)
Visceral	371 (44.8)	323 (46.0)	48 (37.8)
ECOG PS	*n* = 625	*n* = 558	*n* = 67
0	169 (27.0)	161 (28.9)	8 (11.9)
1	323 (51.7)	292 (52.3)	31 (46.3)
2	110 (17.6)	88 (15.8)	22 (32.8)
3	15 (2.4)	10 (1.8)	5 (7.5)
4	8 (1.3)	7 (1.3)	1 (1.5)
Eligible for cisplatin-based chemotherapy	435 (52.5)	399 (56.8)	36 (28.3)
Reason for ineligibility	*n* = 153	*n* = 133	*n* = 20
Creatinine clearance < 60 ml/min	73 (47.7)	66 (49.6)	7 (35.0)
ECOG PS > 2	42 (27.5)	29 (21.8)	13 (65.0)
CTCAE v4, grade ≥ 2 audiometric hearing loss	2 (1.3)	1 (0.8)	1 (5.0)
NYHA class III heart failure	4 (2.6)	2 (1.5)	2 (10.0)
CTCAE v4, grade ≥ 2 peripheral neuropathy	1 (0.7)	1 (0.8)	0 (0.0)
Other	8 (5.2)	6 (4.5)	2 (10.0)
Unknown	41 (26.8)	41 (30.8)	0 (0.0)

Values are number of nonmissing values (%) unless otherwise indicated

*BC* bladder cancer, *CTCAE v4* Common Terminology Criteria for Adverse Events version 4, *ECOG PS* Eastern Cooperative Oncology Group performance status, *NYHA* New York Heart Association, *SD* standard deviation, *UC* metastatic urothelial carcinoma

### Treatment patterns

**Fig. 1:**
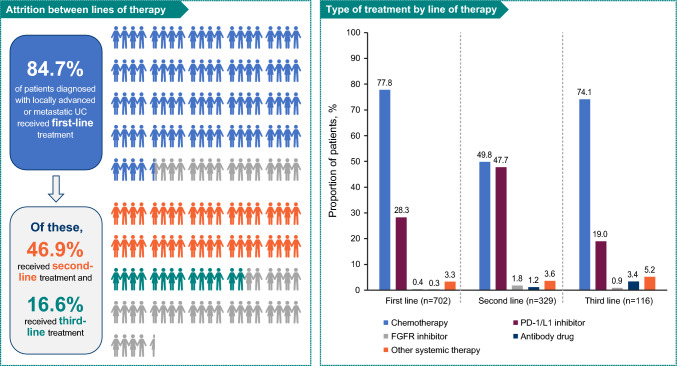
Treatment patterns. *FGFR* fibroblast growth factor receptor, *PD-1/L1* programmed cell death protein 1/ligand 1, *UC* urothelial carcinoma

Most (84.7% [*n* = 702]) patients received first-line therapy. In the first-line setting, 77.8% (*n* = 546) of patients received chemotherapy (chemotherapy alone: 70.2% [*n* = 493]) and 28.3% (*n* = 199) received PD-1/L1 inhibitor therapy (PD-1/L1 inhibitors alone: 18.4% [*n* = 129]) (Supplemental Table 1). Of patients receiving first-line therapy (*n* = 702), 46.9% (*n* = 329) subsequently received second-line therapy and 16.6% (*n* = 116) received third-line therapy (Fig. 1). Chemotherapy was also the most common therapy in the second- and third-line settings, followed by PD-1/L1 inhibitors (Supplemental Table 1).

### Survival and disease progression

**Fig. 2:**
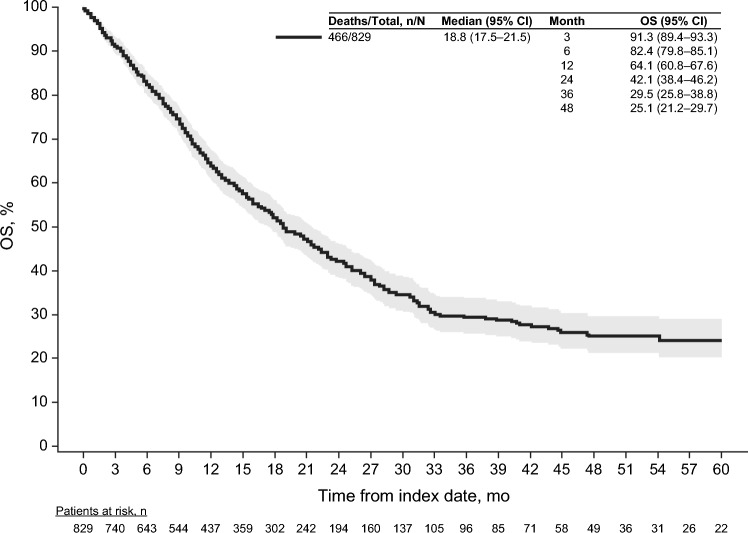
Overall survival from la/mUC diagnosis. *CI* confidence interval, *OS* overall survival

Four hundred and forty-six patients (56.2%) died during the follow-up period (median follow-up, 12.7 months). Overall, estimated median OS from diagnosis of la/mUC was 18.8 (95% confidence interval [CI] 17.5–21.5) months, and the 12-month survival rate was 64.1% (Fig. 2). From the start of each line of therapy, estimated median OS was 16.9 (95% CI 14.3–18.9) months for the first-line setting (*N* = 702), 11.6 (95% CI 9.6–14.3) months for the second-line setting (*N* = 329), and 9.9 (95% CI 7.9–12.6) months for the third-line setting (*N* = 116) (Supplemental Table 2).

**Fig. 3:**
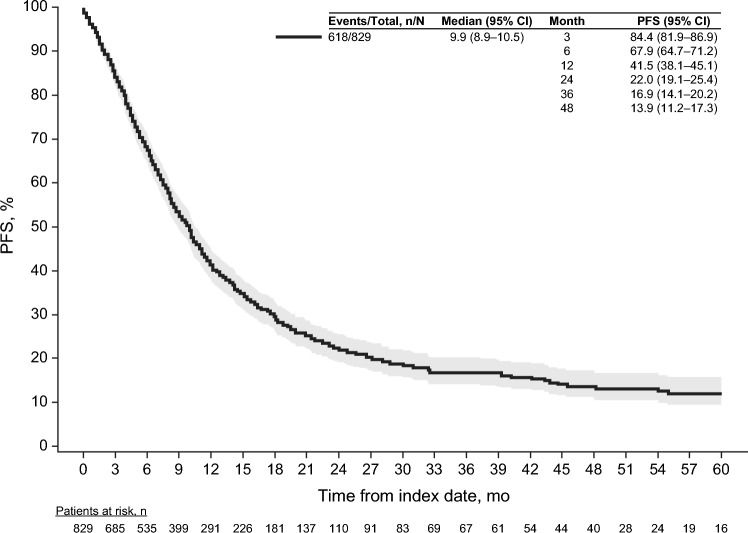
Progression-free survival from la/mUC diagnosis. *CI* confidence interval, *PFS* progression-free survival

Most (74.5% [*n* = 618]) patients in the study population experienced disease progression or death after la/mUC diagnosis during the follow-up period. Median PFS from la/mUC diagnosis was 9.9 (95% CI 8.9–10.5) months and the 12-month PFS rate was 41.5% (Fig. 3). Median time to progression was 12.7 (95% CI 11.3–14.6) months.

### Health care resource utilization

Data on HCRU were available for 649 patients in the study. Most (71.8% [*n* = 595]) patients in the study population had at least one outpatient visit, 56.6% (*n* = 469) had at least one inpatient admission, and 56.5% (*n* = 468) had at least one ED visit associated with la/mUC. Mean (standard deviation [SD]) number of visits per month was 2.2 (4.5) for outpatient visits, 0.4 (1.8) for inpatient admissions, and 0.4 (0.7) for ED visits. Median duration of inpatient admission was 8.0 (range 2.0–23.0) days.

## Discussion

The present study characterizes real-world patient characteristics, treatment patterns, survival, and HCRU among patients with la/mUC at university hospitals in Spain from 2015 to 2020. It provides country-specific, real-world evidence about la/mUC to inform health care professionals about local treatment patterns, survival, and HCRU in Spain during that time period. However, the study period predates European Medicines Agency approvals of avelumab maintenance therapy [8] and enfortumab vedotin for second-line therapy [11]. Nevertheless, as the treatment landscape for la/mUC continues to evolve and real-world data become available for more recently approved therapies, the results of this study provide a historical perspective that could contextualize future results.

Results of our descriptive analysis are comparable with those of a previously published population-based registry study of patients with primary or recurrent bladder cancer in Spain: In the present study and the Miñana et al. [12] study, most patients were men, mean ages at diagnosis were similar, and most patients had a history of smoking. The high percentage of patients (75%) in the present study who had a history of smoking was not unexpected and is consistent with the evidence of tobacco use as a primary risk factor for bladder cancer [13]. Patients included in the present study had a high comorbidity burden, as evidenced by a high mean Charlson Comorbidity Index and the presence of comorbidities such as cardiovascular disease, pulmonary disease, and diabetes. In the Miñana et al. [12] study, most patients had a first diagnosis of noninvasive bladder cancer, whereas a first diagnosis of la/mUC was most common in the present study. This result was not unexpected because the present study was limited to patients with advanced disease and earlier stages can be treated with curative intent. Nevertheless, more than one-half of patients with la/mUC seen in practice in the present study had progressed from noninvasive and muscle-invasive bladder cancers.

The results of our real-world chart review study are also consistent with Milloy et al.’s [14] analysis of 361 patients in Spain using data from the Adelphi metastatic UC Disease Specific Programme, which is a multinational, cross-sectional survey of physicians and their consulting patients [14]. Similar to Milloy et al. [14], we found that therapeutic patterns were consistent with Spanish and international guideline recommendations; that is, most patients received first-line platinum-based chemotherapy. However, our study data are longitudinal in nature and therefore allow us to describe patient attrition between different lines of treatment, to present PFS and OS outcomes, and to describe HCRU. The attrition between lines of therapy and survival observed in our study highlights the need for additional treatments for the la/mUC population.

Results of our study showed that chemotherapy was the preferred first-line treatment among patients with la/mUC, a finding consistent with the recommended use of platinum-based chemotherapy as first-line standard of care during the study period [6]. The frequency of PD-1/L1 inhibitor use observed in the first-line setting in our study population (28.3%) was similar to that observed in a real-world study in the United States (24.1%) [15] but higher than reported in real-world studies in Canada (1.3%) [16] or the Netherlands (8.0%) [17]. Use of PD-1/L1 inhibitors in the first-line setting could have been related to patient access to first-line PD-1/L1 inhibitors and our study population being selected from university referral hospitals, which are often associated with high recruitment for clinical trials, particularly in the context of the high number of clinical trials implemented in Spain in the previous 10 years.

Our results showed an overall median OS of 18.8 (95% CI 17.5–21.5) months and a median PFS of 9.9 (95% CI 8.9–10.5) months from diagnosis of la/mUC among patients in Spain. This is numerically higher compared with recent results of the real-world US studies by Geynisman et al. [15], who reported a median OS of 12.4 (95% CI 12.0–12.9) months and a median PFS of 8.5 (95% CI 8.3–8.7) months from time of la/mUC diagnosis, and Sonpavde et al. [18], who reported a median OS of 11.0 (95% CI 10.3–11.5) months. However, our study was not designed to compare OS outcomes with other data sources, and a direct comparison of OS across different study cohorts is challenging due to differences in patient characteristics that may be prognostic for OS. We reported a difference of about 2 months between median OS measured from la/mUC diagnosis (18.8 months) versus from start of first-line therapy (16.9 months). It is possible that a similar difference could be present if median PFS were calculated from treatment initiation rather than index date.

The increasing cost of cancer care is of great concern for the national health care system. The demographic profile of patients with la/mUC in the present study population and the sequential nature of the treatment plan may be reflected in resource utilization. Most patients included in the present analysis required outpatient appointments and had at least one inpatient admission or ED visit, highlighting the burden for patients with la/mUC.

The retrospective nature of the study is a limitation, primarily because of the use of medical records. Bias could have been introduced through practice pattern variations and study sites being limited to university referral hospitals, which are often associated with high recruitment for clinical trials and may also result in higher treatment rates in subsequent treatment lines. The potential patient selection bias could explain the high use of first-line systemic therapy, which could have subsequently impacted OS rates. The high use of first-line immunotherapy is one potential influence on the HCRU observed in our results.

## Conclusions

Advances in immunotherapy and other mechanism of actions are shifting the treatment landscape for select patients with la/mUC, but innovative treatments that could improve outcomes among a broader, nonrestricted population are still needed. More than one-half of patients in this study had at least one inpatient admission or ED visit. Attrition between lines of therapy observed in this study highlights the need for additional treatments for this population. Studies using real-world data that include cost effectiveness and health-related quality-of-life analyses, as well as studies to determine the value and impact of treatment choice/sequence on patient outcomes and disease burden, are needed. Our results show that therapeutic patterns were consistent with Spanish guideline recommendations, with chemotherapy having a significant role in first-line treatment of la/mUC in Spain during the study period. However, the disease burden was high, and new treatments recommended in the most recent European guidelines as the preferred first-line option should be made available to patients in Spain.

## Data availabity

Researchers may request access to anonymized participant level data, trial level data and protocols from Astellas sponsored clinical trials at www.clinicalstudydatarequest.com. For the Astellas criteria on data sharing see: https://clinicalstudydatarequest.com/Study-Sponsors/Study-Sponsors-Astellas.aspx. Dr Javier Puente had full access to all the data in the study and takes responsibility for the integrity of the data and the accuracy of the data analysis.

## Supplementary Information

Below is the link to the electronic supplementary material.

Supplementary file1 (PDF 182 KB)

## Abbreviations

*ECOG*: Eastern Cooperative Oncology Group
*ED*: Emergency department
*HCRU*: Health care resource utilization
*la/mUC*: Locally advanced or metastatic urothelial carcinoma
*OS*: Overall survival
*PD-1*: Programmed cell death protein 1
*PD-L1*: Programmed death ligand 1
*PFS*: Progression-free survival
*PS*: Performance status
*SD*: Standard deviation
*UC*: Urothelial carcinoma
