# Supplementary material for: Real-world treatment patterns, survival outcomes, and health care resource utilization for locally advanced or metastatic urothelial carcinoma in Spain
Source: Clin Transl Oncol. 2024 Oct 4;27(5):2232–40. doi: 10.1007/s12094-024-03734-8 (PMC12033175; doi:10.1007/s12094-024-03734-8)
Supplement: Supplementary file 1 — Supplementary file1 (PDF 182 KB) [file 12094_2024_3734_MOESM1_ESM.pdf]

Real-World Treatment Patterns, Survival Outcomes, and Health Care Resource Utilization for Locally Advanced or Metastatic Urothelial Carcinoma in Spain

Javier Puente, MD, PhD[a]; Alvaro Pinto, PhD[b]; Maria José Mendez-Vidal, MD[c]; Xavier García del Muro, PhD[d]; Pablo Maroto, PhD[e]; Sergio Vazquez, MD[f]; Raquel Luque-Caro, PhD[g]; Urbano Anido, PhD[h]; Torsten Strunz-McKendry, PhD[i]; Anil Upadhyay, MD[i]; Jose Montes[j]; Aurora Ortiz Nuñez, PhD[i]; Judit González Portela, PharmD[i]; Daniel Castellano, MD[k]

[a]Medical Oncology Department, Hospital Clínico San Carlos, Instituto de Investigación Sanitaria del Hospital Clínico San Carlos (IdISSC), CIBERONC, Madrid, Spain; [b]Servicio de Oncologia Medica, Hospital Universitario La Paz, Madrid, Spain; [c]Medical Oncology Department, Maimonides Institute for Biomedical research of Córdoba (IMIBIC) Hospital Universitario Reina Sofía, Cordoba, Spain; [d]Institut Català d'Oncologia, IDIBELL. University of Barcelona, Spain; [e]Servicio de Oncologia Medica, Hospital de la Santa Creu i Sant Pau, Barcelona, Spain; [f]Servicio de Oncología Médica. Hospital Universitario Lucus Augusti, Lugo, Spain; [g]Servicio Oncología Médica, Hospital Universitario Virgen de las Nieves, Instituto de Investigación Biosanitaria ibs. Granada, Granada, Spain; [h]Servicio de Oncologia Medica, Hospital Universitario de Santiago, Santiago, Spain; [i]Astellas Pharma Europe Ltd, Addlestone, Surrey, UK; [j]Effice Research, Madrid, Spain; [k]Medical Oncology Department, Hospital Universitario 12 de Octubre, Madrid, Spain

Corresponding Author:

Javier Puente, MD, PhD

Medical Oncology Department

Hospital Clínico San Carlos, Instituto de Investigación Sanitaria del Hospital Clínico San Carlos (IdISSC), CIBERONC

Calle del Prof Martín Lagos, S/N, 28040

Madrid, Spain

Phone: +34 913 30 30 00

Fax: +34 913 30 30 00

Email: javierpuente.hcsc@gmail.com

ORCiD: 0000-0002-6910-1331

SUPPLEMENTARY INFORMATION

SUPPLEMENTAL FIGURE

Supplemental Fig. 1 Study schematic. [a]Date of first diagnosis/record of locally advanced or metastatic urothelial carcinoma

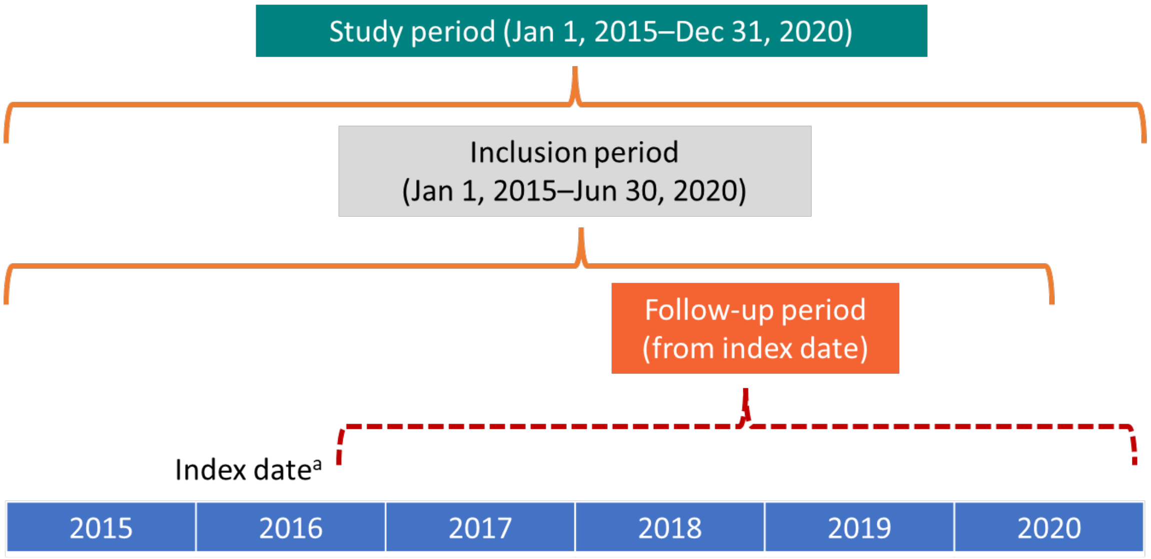

SUPPLEMENTAL TABLES

Supplemental Table 1 Treatment patterns of locally advanced or metastatic urothelial carcinoma by line of therapy

Treatment	Line of therapy First (n = 702)	Second (n = 329)	Third (n = 116)
Median (IQR) treatment duration, months	3.2 (1.6, 4.9)	2.3 (1.2, 4.2)	2.7 (1.4, 4.7)
Treatment type			
Chemotherapy alone	493 (70.2)	153 (46.5)	84 (72.4)
PD-1/L1 inhibitor therapy alone	129 (18.4)	147 (44.7)	21 (18.1)
Other monotherapies	9 (1.3)	15 (4.6)	8 (6.9)
Chemotherapy + PD-1/L1 inhibitor combination therapy	52 (7.4)	7 (2.1)	0 (0.0)
Other combination therapy	19 (2.7)	7 (2.1)	3 (2.6)
Chemotherapy[a]	546 (77.8)	164 (49.8)	86 (74.1)
Platinum-based therapy	509 (93.2)	90 (54.9)	17 (19.8)
Carboplatin-based	322 (63.3)	73 (81.1)	11 (64.7)
Cisplatin-based	186 (36.5)	16 (17.8)	6 (35.5)
Oxaliplatin-based	1 (0.2)[b]	1 (1.1)[c]	NA
Vinflunine	11 (2.0)	46 (28.0)	37 (43.0)
Gemcitabine	7 (1.3)	2 (1.2)	NA
Docetaxel	4 (0.7)	15 (9.1)	16 (18.6)
Paclitaxel	3 (0.5)	9 (5.5)	13 (15.1)
Other chemotherapy	12 (2.2)	2 (1.2)	3 (3.5)
PD-1/L1 inhibitor therapy[a]	199 (28.3)	157 (47.7)	22 (19.0)
Atezolizumab	102 (51.3)	117 (74.5)	16 (72.7)
Pembrolizumab	28 (14.1)	14 (8.9)	1 (4.5)
Durvalumab	18 (9.0)	5 (3.2)	NA
Nivolumab	17 (8.5)	6 (3.8)	3 (13.6)
Avelumab	14 (7.0)	13 (8.3)	1 (4.5)
Nivolumab plus ipilimumab	1 (0.5)	2 (1.3)	1 (4.5)
Other PD-1/L1 inhibitor	19 (9.5)	NA	NA
FGFR inhibitor[a]	3 (0.4)	6 (1.8)	1 (0.9)
Antibody drug[a]	2 (0.3)	4 (1.2)	4 (3.4)
Other systemic therapy[a]	23 (3.3)	12 (3.6)	6 (5.2)

Values are n (%)

FGFR fibroblast growth factor receptor, *NA* not applicable, *PD-1/L1* programmed cell death protein 1/ligand 1

[a]Treatment types are not mutually exclusive

[b]Capecitabine + oxaliplatin

[c]Folinic acid + fluorouracil + oxaliplatin (FOLFOX)

Supplemental Table 2 Overall survival from start of treatment line by line of therapy

Survival	Line of therapy<br>First (n = 702)	Second (n = 329)	Third (n = 116)
Death	390 (55.6)	195 (59.3)	73 (62.9)
Alive	312 (44.4)	134 (40.7)	43 (37.1)
Median (95% CI) OS, months	16.9 (14.3–18.9)	11.6 (9.6–14.3)	9.9 (7.9–12.6)

Values are n (%) unless otherwise indicated
CI confidence interval, *OS* overall survival
